# Topological Surface States in a Gyroid Acoustic Crystal

**DOI:** 10.1002/advs.202205723

**Published:** 2022-12-16

**Authors:** Yuning Guo, Matheus I. N. Rosa, Massimo Ruzzene

**Affiliations:** ^1^ P. M. Rady Department of Mechanical Engineering University of Colorado Boulder Boulder CO 80309 USA

**Keywords:** gyroid surface, nonsymmorphic symmetry, phononic semimetal state, surface mode

## Abstract

The acoustic properties of an acoustic crystal consisting of acoustic channels designed according to the gyroid minimal surface embedded in a 3D rigid material are investigated. The resulting gyroid acoustic crystal is characterized by a spin‐1 Weyl and a charge‐2 Dirac degenerate points that are enforced by its nonsymmorphic symmetry. The gyroid geometry and its symmetries produce multi‐fold topological degeneracies that occur naturally without the need for ad hoc geometry designs. The non‐trivial topology of the acoustic dispersion produces chiral surface states with open arcs, which manifest themselves as waves whose propagation is highly directional and remains confined to the surfaces of a 3D material. Experiments on an additively manufactured sample validate the predictions of surface arc states and produce negative refraction of waves at the interface between adjoining surfaces. The topological surface states in a gyroid acoustic crystal shed light on nontrivial bulk and edge physics in symmetry‐based compact continuum materials, whose capabilities augment those observed in ad hoc designs. The continuous shape design of the considered acoustic channels and the ensuing anomalous acoustic performance suggest this class of phononic materials with semimetal‐like topology as effective building blocks for acoustic liners and load‐carrying structural components with sound proofing functionality.

## Graphical abstract

The work reports a 3D gyroid acoustic crystal hosting multi‐fold degeneracies associated with topological surface states. The unique chiral morphology of the gyroid surface is leveraged to inherently provide this 3D acoustic material with non‐trivial dispersion topology, revealing the continuous design can ensure anomalous acoustic performance and remarkable functionalities without ad hoc designs.

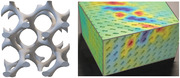

## 1 Introduction

Triply periodic minimal surfaces have attracted significant interest in the design of structured materials that leverage their complex morphologies and symmetries to achieve superior mechanical properties. Their architecture provides an efficient tessellation of space resulting in materials with high stiffness and strength,^[^ ^1^, ^2^, ^3^, ^4^, ^5^, ^6^ ^]^ while also incorporating additional functionalities such as frequency band gaps,^[^ ^7^ ^]^ topological waveguiding,^[^ ^8^ ^]^ energy absorption,^[^ ^9^ ^]^ and thermal management,^[^ ^10^ ^]^ among others. The gyroid surface is endowed by a spiral‐like shape with cubic symmetry and chiral morphology, whose properties have been explored in various fields including biology, mechanics, optics, and acoustics.^[^ ^11^, ^12^, ^13^, ^14^, ^15^, ^16^, ^17^, ^18^, ^19^, ^20^ ^]^ The observation of gyroid morphologies in nature and self‐assembly systems also suggests that its architecture is mechanically robust.^[^ ^20^, ^21^, ^22^ ^]^ In addition, materials based on gyroid surfaces applied as photonic crystals have shown extraordinary optical properties such as complete bandgaps, linear/circular dichroism, and Weyl points and line nodes.^[^ ^17^, ^18^, ^23^, ^24^, ^25^ ^]^ Despite these achievements, the potential of gyroid structured materials for topological wave physics phenomena has not been fully explored in the context of acoustics and elastic waves.

We here investigate an acoustic gyroid crystal and exploit the non‐trivial acoustic dispersion topology that naturally occurs due to its unique geometry and symmetry. The ensuing topological surface states have not yet been demonstrated in 3D continuous material platforms such as minimal surface‐based architectures. In parallel to the development of gapped phases of topological insulators,^[^ ^26^, ^27^, ^28^, ^29^ ^]^ gapless phases have been recently observed in the so‐called “topological semimetals,”^[^ ^30^, ^31^, ^32^ ^]^ based on which classical analogues have been explored in both photonic and phononic systems.^[^ ^24^, ^33^, ^34^, ^35^, ^36^, ^37^, ^38^, ^39^, ^40^, ^41^ ^]^ These semimetals are characterized by band structures with multi‐fold band degeneracies points like Weyl and Dirac points, or other touching patterns like nodal lines and nodal rings, which do not require a band gap and can result in Fermi arcs associated with topologically protected surface states.^[^ ^42^, ^43^, ^44^ ^]^ Recent studies illustrate that multi‐fold topological band degeneracies are protected by crystalline space‐group symmetries in electric, photonic, and phononic systems.^[^ ^40^, ^41^, ^45^, ^46^ ^]^ Two established configurations for phononic semimetal states are based on layer‐stacked honeycomb lattices with proper interlayer coupling,^[^ ^37^, ^38^ ^]^ and cubic lattices with nonsymmorphic symmetries.^[^ ^39^, ^40^, ^41^ ^]^ These notable studies advance the state of the art by implementing topological surface states in phononics, however they rely on demonstrator models with ad hoc designs that are not readily applicable as compact continuous materials. Also, minimal surface‐based materials were shown to integrate efficient load‐bearing capabilities with the robust topological waveguiding features of gapped insulators,^[^ ^8^ ^]^ and therefore may provide a path toward continuous materials hosting gapless topological states.

Herein, we design a 3D gyroid acoustic crystal that hosts multi‐fold topological degenerate points which are associated with the existence of topological surface states. Indeed, the gyroid surface is a natural candidate for exploring non‐trivial dispersion topology within a continuous material platform due to its nonsymmorphic symmetry. Our results illustrate the emergence of a spin‐1 Weyl and a charge‐2 Dirac degenerate points, whose topological character is connected to chiral topological states that propagate confined to the surfaces of the material. In addition to surface confinement, the topological states define open arcs in the reciprocal space, which makes their propagation along the surfaces highly directional. These topological states are experimentally observed on an additive manufactured sample, illustrating the ability to control the propagation of waves at the surface of the material. Our experiments also evidence how the directional wave properties resulting from surface arcs in reciprocal space produce the negative refraction of surface modes propagating across the interface between two neighboring facets of the 3D material. These features are naturally enforced by the nonsymmorphic symmetry of the gyroid, revealing that gapless topological states can widely exist in this class of continuous materials without fine parameter tuning or delicate symmetry designs, which do not provide self‐contained material platforms. The proposed gyroid embedded with a rigid material provides the framework for the design of an acoustic material that leverages topological waveguiding and directional propagation which may translate into sound redirection and insulation capabilities.

## 2 Geometry of Gyroid Acoustic Crystals

**Figure 1:**
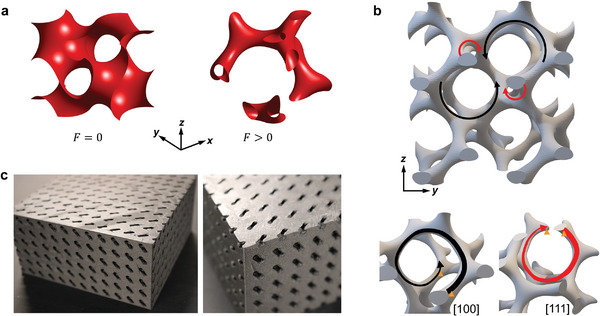
Geometry of gyroid acoustic crystals. a) Unit cells of gyroid surface with *F* = 0 and *F* > 0. b) Illustration of the inherent chirality of the gyroid surface. The red and black spiral lines indicate the left‐handed helices and right‐handed helices, respectively. c) Photographs of the 3D printed sample of gyroid acoustic crystal which incorporates airborne gyroid channels.

Crystal symmetry plays a critical role in the physics of topological materials. In this context, 3D band degenerate points introduced by the nonsymmorphic symmetries provide an effective approach to realizing 3D semimetal states through a combination of point group symmetry and translation of a Bravais lattice vector.^[^ ^47^, ^48^ ^]^ The single gyroid, a triply periodic minimal surface with body‐centered cubic symmetry, belongs to the nonsymmorphic space I4_1_32 (No. 214, a subgroup of $Ia\bar{3}d$). It features multiple rotational axes and screw axes as well as numerous axial and diagonal glide planes, but does not have any mirror symmetry, and therefore it is chiral in nature.^[^ ^18^, ^46^, ^49^, ^50^, ^51^ ^]^ The gyroid can be defined by the isosurface function *F* = cos (*x*) sin (*y*) + cos (*y*)sin (*z*) + cos (*z*)sin (*x*), where the parameter *F* defines the morphology type. For 0 ≤ |*F*| < 1, the gyroid forms a single surface that partitions the space into two regions as illustrated by the *F* = 0 example in the left panel of **Figure** **1**a. In the range 1 < |*F*| < 1.413, the gyroid surfaces define closed channels with open ports at the boundaries as illustrated by the *F* = 1.08 example in the right panel of Figure 1a.^[^ ^1^, ^17^, ^52^ ^]^ In this work, the value *F* = 1.08 is utilized to define the geometry of gyroid channels within a rigid material. This produces an acoustic crystal, within which spiral channels support the airborne propagation of acoustic waves. Local helices along the screw axes of the gyroid can be identified in different directions, e.g., [100], [001], or [111].^[^ ^17^, ^53^ ^]^ The chirality of a gyroid surface in the *yz* plane is illustrated in Figure 1b, where left‐handed helices and right‐handed helices are highlighted. The zoomed view illustrates the chiral path that connects two points (orange triangular markers) translated by a unit cell along the [100] and [111] directions, respectively. Figure 1c shows perspective views of the fabricated 3D sample of gyroid acoustic crystal, whereby the solid material occupies the region that is complementary to the airborne gyroid channels. The sample consists of 12 × 12 × 6 unit cells in a cubic lattice structure with the lattice spacing *a* = 10 mm, and is fabricated through fused deposition modeling using a Markforged printing machine (see Experimental Section). Considering the large acoustic impedance mismatch between the solid material and the air, the acoustic‐solid boundaries are treated as rigid, and sound propagates only through the gyroid channels. The results that follow focus on the choice *F* = 1.08 as a demonstration, but similar properties are found in the entire regime 1 < |*F*| < 1.413 since it results in similar morphologies.

## 3 Results

**Figure 2:**
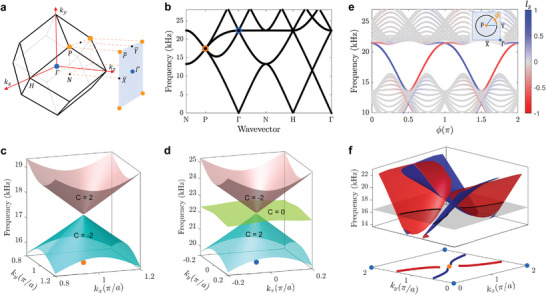
Dispersion properties of the acoustic gyroid crystal. a) 3D Brillouin zone (BZ) of the gyroid surface and its projected surface BZ at the *xy* plane. The dots with orange color and blue color denote the symmetry points *P* and *Γ* in the 3D BZ, and also the corresponding projected symmetry points $\bar{P}$ and $\bar{\Gamma }$ in the surface BZ. b) Bulk dispersion along the high symmetry lines of the 3D BZ. c,d) 3D plot of the surface dispersion in the vicinity of the quadruple degenerate point and triple degenerate point, respectively. The Chern number of each band is labeled. e) Surface dispersion along a circular momentum loop centered at $\bar{P}$. The inset shows the circular loop of radius 0.75*π*/*a* centered at $\bar{P}$ in surface BZ. The branches are color‐coded based on the polarization of the corresponding modes as defined by the localization factor *l*_s_, where blue and red colors denote modes localized at the top and bottom surfaces, respectively. f) Dispersion of surface states in the surface BZ centered at $\bar{P}$ (upper panel). The isofrequency contour with a frequency of 16.5 kHz (bottom panel) acts as a demonstration of open surface arcs. The surface states share the same color code with (e).

We first investigate the dispersion and wave propagation properties of the gyroid acoustic crystal through numerical simulations conducted within the COMSOL Multiphysics software (see Experimental Section for details on numerical simulations). **Figure** **2**a shows the 3D body‐center cubic Brillouin zone (BZ), and a projected surface BZ for the considered gyroid. The 2D surface BZ is obtained by projecting the 3D BZ onto a specific plane, the *xy* plane in the following discussion. The orange and blue dots denote the positions of the symmetry points *P* and *Γ* in the 3D BZ, and the corresponding projected symmetry points $\bar{P}$ and $\bar{\Gamma }$ in the surface BZ. The bulk dispersion of the acoustic gyroid material of geometry and size corresponding to the experimental sample shown in Figure 1c is displayed in Figure 2b along the high symmetry lines of the 3D BZ. It exhibits a fourfold degenerate band crossing at the high‐symmetry point *P* (the four bands consisting of two sets of doubly degenerate bands), and a three‐fold degenerate band crossing at *Γ*. These degenerate nodal points are protected by the nonsymmorphic symmetry of the gyroid surface.^[^ ^45^, ^48^ ^]^ Here we focus on the properties of the symmetry‐enforced degeneracies at *P* and *Γ* within 13–28 kHz, but there are other band degenerate points appearing at a higher frequency range (34–48 kHz) or other symmetry lines like the *Γ* *H* line, which may also be considered in future studies.

Figure 2c,d shows the surface dispersion on the *k*_x_ *k*_y_ plane in the vicinity of the quadruple and triple degenerate points at *P* and *Γ*, where linear dispersions are observed around the degeneracies. The non‐trivial topological character of the bands is evidenced by their Chern numbers, which are computed from the evolution of the Wannier centers around closed loops in the 3D BZ by using the Wilson loop method^[^ ^40^, ^54^, ^55^ ^]^ (see Supporting Information for details). The Chern numbers of the two doubly degenerate bands in Figure 2c are calculated as −2 and +2, signaling the degeneracy at *P* is a charge‐2 Dirac point.^[^ ^56^, ^57^, ^58^ ^]^ The four eigenmodes at the charge‐2 Dirac point define two sets of modes with opposite chirality, and the bands crossing at this point exhibit linear dispersion in all directions in the 3D momentum space, which further confirms that this quadruple degenerate point is a Dirac point (see Supporting Information for details). Meanwhile, the three bands in the vicinity of the triple degenerate point at *Γ* exhibit Chern numbers of −2, 0, and +2, respectively, which indicates that this degenerate point is a threefold spin‐1 Weyl point.^[^ ^58^, ^59^, ^60^ ^]^ This deduction is also evidenced by eigenmodes of distinct chirality and the linear dispersions crossing at this point (see Supporting Information for details). The charge‐2 Dirac point and spin‐1 Weyl point are enforced by the nonsymmorphic symmetry of the gyroid material, illustrating that multi‐fold degeneracies with Chern numbers >1 are naturally occurring in this class of continuous materials without requiring intricate ad hoc designs.^[^ ^42^, ^61^, ^62^ ^]^

According to the bulk‐edge correspondence principle, a degeneracy with non‐zero topological results in surface states that can be identified along a closed loop encircling the degeneracy in the projected surface BZ.^[^ ^35^ ^]^ This is illustrated in Figure 2e, which depicts the 2D surface dispersion along a circular loop of radius 0.75*π*/*a* centered at $\bar{P}$ (as the inset indicates). The dispersion is obtained by using a ribbon structure consisting of 1 × 1 × 8 cells with rigid boundaries along the *z* direction, and periodic boundary conditions applied along the *x* and *y* directions. Surface states are identified by computing a localization factor *l*_s_ for each mode. The localization factor is defined based on the ratio between the integral of the pressure field at the surfaces and the integral across the entire ribbon, indicating a surface mode when its value approaches ±1, or a bulk mode when it approximates zero (see Supporting Information for details). The dispersion branches of the ribbon are color‐coded according to *l*_s_, whereby surface modes localized at the top and bottom surfaces are respectively associated with positive and negative values (blue and red colors), while bulk modes are associated with *l*_s_ close to zero (gray color). Four chiral surface bands are identified, two with positive slope corresponding to modes localized at the bottom surface, and two with negative slope corresponding to modes localized at the top surface. This behavior is expected from the degenerate charge‐2 Dirac point with − 2/ + 2 Chern numbers. These surface modes also circle around $\bar{\Gamma }$, for completeness shown in a separate plot in the Supporting Information. We note that the gyroid acoustic crystal supports chiral surface states regardless of the type of surface termination as enforced by the topological degeneracy, but the dispersion of the surface states may be different under different termination types (see Supporting Information for details). The sensitivity with respect to surface terminations provides an additional degree of freedom which opens the possibility of tailoring the surface states on 3D bulk materials.^[^ ^16^ ^]^

The dispersion of the identified surface modes is displayed in the upper panel of Figure 2f along the entire surface BZ in the projected *k*_x_ *k*_y_ plane. The four sheets of the surface states surround the degenerate point, spanning a broad frequency range with a relative bandwidth Δ*ω*/*ω* of about 45%. The contours of the surface states at any frequency define open arcs analogous to the well‐known Fermi arcs of electronic systems.^[^ ^63^, ^64^ ^]^ An example of the open arcs at a frequency of 16.5 kHz is highlighted in the surface plot and displayed in the bottom panel of Figure 2f. Their chiral nature implies that only one pair out of the four arcs are defined at a single surface (the other pair being localized at the opposite surface), which makes their propagation through the specific surface highly directional. Naturally, while the *xy* plane was selected to exemplify the nature of the surface states, these would also exist in other planes such as *xz* or *yz* planes, which we will later exploit to achieve negative refraction through states of different surfaces.

**Figure 3:**
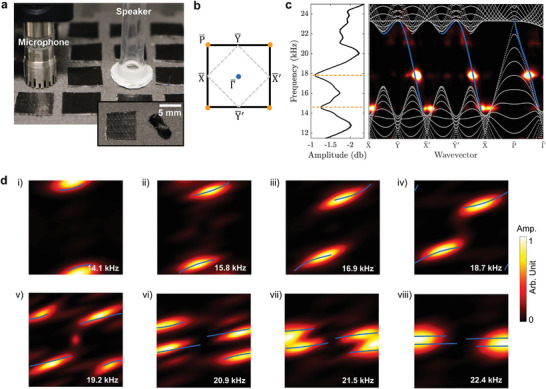
Experimental observation of surface states. a) Measurement setup of the acoustic field at the top *xy* surface of the sample. The holes of airborne channels at a surface to be mapped are sealed by duct tape, except for a reserved hole around the center of the surface for signal excitation and a hole for signal detection. b) The surface BZ centered at $\bar{\Gamma }$. c) Measured average surface transmission (left) and the surface dispersion along the high‐symmetry lines (right). The gray dots and the blue fitted line in the surface dispersion denote numerically predicted bulk modes and the surface modes, respectively. d) Experimental observation of surface arcs for various frequencies. The blue lines denote numerically predicted surface arcs.

Experimental measurements are performed to identify the topological surface states, which are unveiled by the acoustic surface field mapping and associated Fourier spectra. **Figure** **3**a shows the measurement setup for mapping the acoustic field at the top surface of the sample. To excite the surface states, a broadband acoustic signal is fed to a speaker connected to a subwavelength‐sized tube that is placed at a hole at the center of the sample surface. The acoustic pressure is measured hole‐by‐hole by a ¼ inch microphone (GRAS type BD 46) and recorded by a dynamic signal analyzer (see Experimental Section). To mimic the rigid boundary conditions considered in the simulations, the other holes on the upper surface are sealed with duct tape, except for the excitation and measurement points. The photo showing the detail of a sealed hole and an unsealed hole is displayed in the inset of Figure 3a.

The measured pressure field *p*(*x*, *y*, *t*) is transformed to its reciprocal space representation $\hat{p}\left(k_{x},k_{y},\omega \right)$ through a 3D Fourier transformation which produces a representation of the wavefield in the frequency/wavenumber domain.^[^ ^65^ ^]^ Figure 3b specifies the surface BZ used in the analysis of the experimental results, which maintains the projection at the *k*_x_ *k*_y_ plane but with the center shifted from $\bar{P}$ to $\bar{\Gamma }$. Figure 3c compares the measured transmission spectra (colormap) to the numerical surface dispersion along the specific momentum path indicated in Figure 3b. The gray dots correspond to bulk modes, while the blue line denotes the chiral surface modes localized at the top surface. The measured pressure field confirms the excitation of the chiral surface states predicted by the numerical simulations. The left panel in Figure 3c shows the frequency spectrum of the pressure field averaged across the top surface, where the response amplitude varies with frequency considering the acoustic resonances in the sample. The transmission peaks indicated by the dashed lines coincide with the frequencies highlighted in the colormap of the right panel. The matching of the high amplitude regions between the two panels occurs since the average transmission at the surface coincides with the average taken in reciprocal space coordinates *k*_x_,*k*_y_ via a *L*_2_ norm due to Parseval's theorem of Fourier Transforms. Isofrequency contours of the surface dispersion at selected frequencies illustrate the surface arcs: eight representative experimentally observed surface arcs are displayed in Figure 3d at the frequencies of 14.1, 15.8, 16.9, 18.7, 19.2, 20.9, 21.5, and 22.4 kHz, respectively. The colorbar ranges are adjusted for each subfigure for better visualization of the wavefield in the wavenumber domain. We note that for increasing frequencies, the surface arcs in the surface BZ progressively rotate and align along the horizontal direction, which is in good agreement with the numerical predictions (blue lines). The open surface arcs are indicative of directional propagation of the acoustic surface modes, which are further explored in the following.

**Figure 4:**
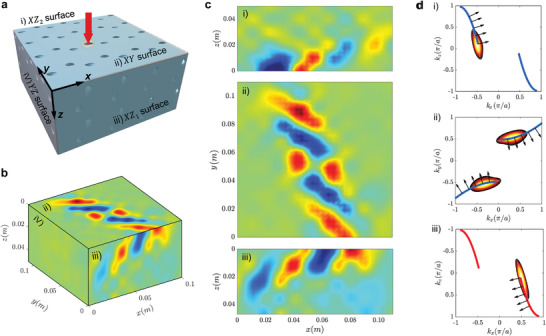
Experimental observation of acoustic negative refraction on the surfaces. a) Schematic of excitation configuration and measured surfaces considered for refraction tests: i) *XZ*_2_ surface, ii) *XY* surface, iii) *XZ*_1_ surface, and iv) *YZ* surface are labeled for reference. b) Measured acoustic fields at 18.4 kHz. c) Representation of measured acoustic fields at 18.4 kHz on the considered surfaces showing negative refraction at surface interfaces. d) Reciprocal space representation of measured acoustic fields (contours), and comparison with theoretical isofrequency lines corresponding to the arcs (solid lines): the arrows represent the directions of the group velocity that are normal to the isofrequency contours.

The topological surface arcs give rise to negative refraction across the surfaces of the sample.^[^ ^40^, ^66^ ^]^ The excitation configuration for the recording of the acoustic field at the frequency where negative refraction is expected is shown in **Figure** **4**a. For the experiments, the holes on the surfaces to be measured are sealed to mimic hard boundary conditions, and a source is fixed at the center of the *XY* surface. The analysis here involves the surface acoustic fields on the *XY*, *YZ*, *XZ*_1_, and *XZ*_2_ surfaces, which are measured through the same procedures and excitation conditions as in the experimental analysis of Figure 3, but with hole‐by‐hole scanning of all surfaces of interest. Figure 4b provides a representation of the experimentally measured acoustic fields in 3D at a frequency of 18.4 kHz, while Figure 4c shows the separate fields at the i) *XZ*_2_, ii) *XY*, and iii) *XZ*_1_ surfaces. The results illustrate the confinement of the acoustic fields to the *XZ*_2_, *XY*, and *XZ*_1_ surfaces according to the excitation of the surface states, while the field at the *YZ* surface confirms the lack of penetration into the bulk of the sample due to the surface‐confined source that excites mostly surface states. Also, the wave propagation at the *XY* surface is highly directional as predicted by its open surface arcs, and exhibits negative refraction as it propagates into the *XZ*_1_ and *XZ*_2_ surfaces. This behavior is further illustrated by the contours shown in Figure 4d representing the measured acoustic fields in the reciprocal space (sharing the same colormap with Figure 3d), which are overlaid to and agree with the numerically predicted isofrequency contours (blue and red lines). The arrows pointing along the normal directions to the contours in Figure 4d indicate the directions of group velocity, which correspond to the directions along which waves propagate at that frequency.^[^ ^67^ ^]^ The surface arcs exhibit small curvatures for a wide range of wavevectors, allowing the acoustic waves to propagate nearly in the same direction as Figure 4c shows. Due to the body‐centered cubic symmetry of the gyroid surface, the surface arcs in the surface BZs at the *xy*, *yz*, and *xz* planes are the same, but rotated in the reciprocal space. Indeed, the surface arcs at the *XZ* and *YZ* surfaces are identical to the surface arcs observed at the *XY* surface, with their isofrequency contours rotated by 90° as shown in Figure 4d (considering a fixed perspective with *k*_x_ as the *x*‐axis). The source at the center of the *XY* surface excites the chiral surface modes with two opposite propagation directions (upward and downward propagation), which can be interpreted from the directions of group velocities demonstrated in the corresponding wavevector space (subfigure (ii) in Figure 4d). When the generated acoustic wave reaches the interface between two adjoining surfaces, it propagates in the neighboring surface according to its surface arc which is rotated by 90°, leading to negative refraction. The non‐zero topological charges of the multi‐fold degenerate points in this design guarantee the existence of the open arcs, ensuring the observation of negative refraction of topologically protected acoustic waves. The degree of surface localization is characterized in the Supporting Information to illustrate the influence of the number of unit cells on the surface states.

## 4 Conclusions

In this work, we investigate a gyroid acoustic crystal that implements a phononic “semimetal” phase. The chiral morphology and nonsymmorphic symmetry of the gyroid surface lead to topological degeneracies and chiral surface states, which are predicted numerically and confirmed experimentally. The topological states are not only confined to the surfaces but are also characterized by highly directional propagation and negative refraction when traversing an edge separating two surfaces of the crystal. We demonstrate these properties on a rigid material block that incorporates gyroid acoustic channels. These features rely solely on the symmetry of the gyroid, which makes the design easily scalable to other length scales and operating frequencies. Our results pave the way to exploiting the observed topological phases of matter for acoustic materials that have the ability to confine sound according to surface states, and that are characterized by anomalous (negative) refraction as the sound propagates across edges of a solid with gyroid channels. These characteristics may prove useful in enhancing the absorption of incident sound, or in reducing the transmission of sound across an acoustic panel design with the considered gyroid channels tuned for a frequency range of interest.

## 5 Experimental Section

### Simulations

The geometry of the gyroid acoustic crystal was constructed by considering the isosurface function in MATLAB with a given lattice constant and *F* value, and then exporting it as a .stl file to the COMSOL Multiphysics software for the following modeling and simulation. All simulations were conducted in the “Pressure Acoustics” module of COMSOL Multiphysics. The gyroid channels were filled with air and only the acoustic wave propagating in the air was considered in the simulation. The density and the acoustic velocity of the air were 1.2 kg m^−3^ and 343 m s^−1^, respectively. For the bulk dispersion obtained from the unit cell, periodic boundary conditions were applied in all three directions. For the surface dispersion in the surface Brillouin zone, a ribbon structure with 1 × 1 × 8 cells was used and periodic boundary conditions were imposed along the *x* and *y* directions, while rigid boundary conditions were applied along the *z* direction. Surface states were identified from the bulk states by inspecting the surface localization of the eigenstates, as described in the Supporting Information.

### Experiments

The physical test sample of the gyroid acoustic crystal was 3D‐printed through fused deposition modeling using a Markforged printing machine and the Onyx material. The size of this acoustic sample was 120 mm × 120 mm × 60 mm. A broadband sound signal with a sweeping frequency of 10–26 kHz was launched from a deep subwavelength tube (inner diameter 4 mm, ≈0.2*λ*_acoustic_) to experimentally excite the surface states. The distribution of the acoustic pressure field was manually measured through a portable probe microphone (GRAS type 46BD) with a radius of 3.2 mm. In the measurements, the microphone was moved to scan the 2D acoustic surface field point by point, where the scanning steps were given by the lattice spacing of 10 mm. The amplitude and phase of the acoustic pressure field were recorded by a Data Translation DT9857E signal analyzer. The recorded signals were then post‐processed within the Matlab environment.

## Conflict of Interest

The authors declare no conflict of interest.

## Supporting information

Supporting Information

## Data Availability

The data that support the findings of this study are available from the corresponding author upon reasonable request.
