# Supplementary material for: Topological Surface States in a Gyroid Acoustic Crystal
Source: Adv Sci (Weinh). 2022 Dec 16;10(6):2205723. doi: 10.1002/advs.202205723 (PMC9951337; doi:10.1002/advs.202205723)
Supplement: Supplementary file 1 — Supporting Information [file ADVS-10-2205723-s001.pdf]

Supplementary Material

Topological Surface States in a Gyroid Acoustic Crystal

*Yuning Guo, Matheus Rosa, Massimo Ruzzene**

S1. Calculation of topological charges

S2. Multifold degenerate points in a gyroid acoustic crystal

S3. Surface dispersions in terms of surface termination

S4. Numerical observation of acoustic surface modes

S1. Calculation of topological charges

The topological charge is a topological invariant of 3D semimetal phases, which in theory can be obtained by integrating the Berry curvature over a small sphere enclosing a band degenerate point. In numerical simulations, the topological charge is usually estimated based on the Wilson loop method by obtaining the Chern number which is equal to the winding number of the Wannier centers.[1-4] For 3D crystals, a 2D Brillouin zone is defined by fixing a certain wavevector component k_i, and the Wannier center is computed along a closed loop path k_j defined based on the two remaining wavevector components, which is expressed as:

$$\mathrm{W}(k_i) = -\mathrm{Im}\left(\log \prod_{k_j} \left\langle u_{(k_i,k_j)}(\boldsymbol{r}) \middle| u_{(k_i,k_{j+1})}(\boldsymbol{r}) \right\rangle\right) \quad \text{(S1)}$$

where $u(\boldsymbol{r})$ is the wavefunction at position $\boldsymbol{r}$ in the unit cell for a given band, and k_j is the parametrized wavevector component along the loop.

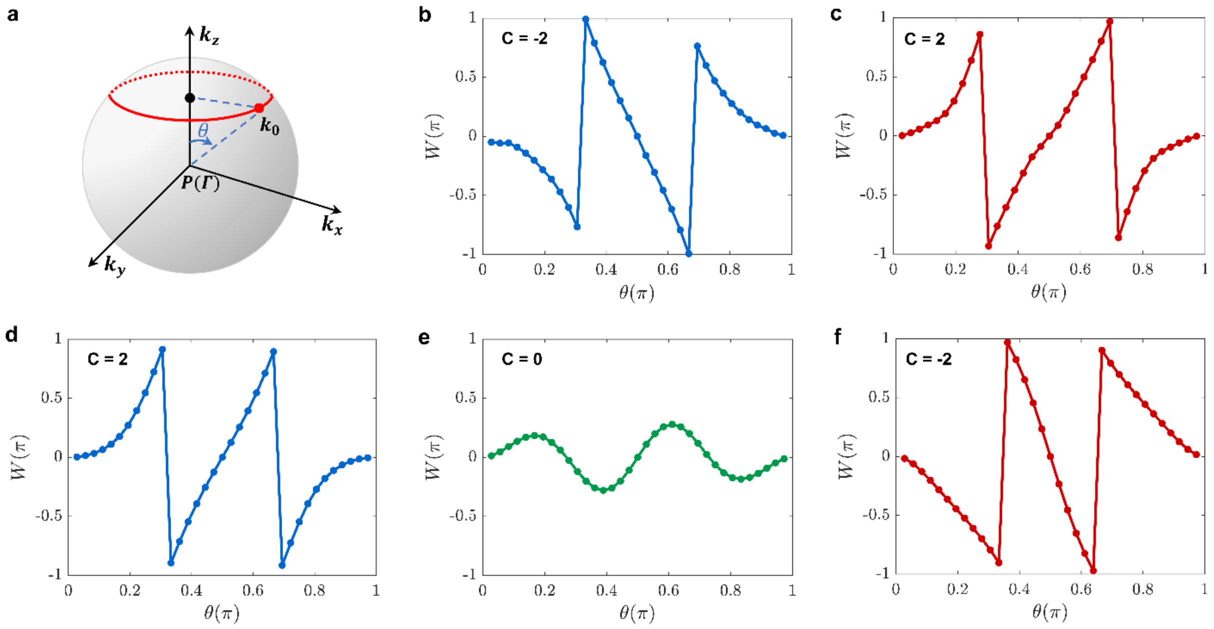

Figure S1. The evolution of the Wannier centers. a) The sphere in the 3D Brillouin zone centered at the high symmetry points P or Γ. b,c) Wannier center evolution of the doubly degenerate first and second bands, third and fourth bands around point P, respectively. d-f) Wannier center evolution of the first, second, and third bands around point Γ, respectively.

In our calculation, the sphere in momentum space is discretized into a sequence of horizontal loops defined at fixed k_z values, parametrized through the relation $k_z = k_0 \cos(\theta)$, where $k_0 = 0.5\,\pi/a$ ($a = 10\ mm$ is the lattice spacing of the gyroid acoustic crystal) is the radius of the sphere in momentum space, and θ is the polar angle in spherical coordinates (**Figure S1a**). The Wannier center along each circular loop $\boldsymbol{k}_\perp\ (k_x, k_y)$ of radius $k_0 \sin(\theta)$ is calculated using the wavefunctions obtained from numerical simulations by using Equation S1. The wavefunctions $u_{\boldsymbol{k}_\perp}(\boldsymbol{r})$ are obtained as $u_{\boldsymbol{k}_\perp}(\boldsymbol{r}) = p_{\boldsymbol{k}_\perp}(\boldsymbol{r})\exp(i\boldsymbol{kr})$, where $p_{\boldsymbol{k}_\perp}(\boldsymbol{r})$ is the pressure value at the position $\boldsymbol{r}$ extracted from the eigenvectors in COMSOL simulations, and $\boldsymbol{k} = (\boldsymbol{k}_\perp, k_z)$. For the degenerate bands, the Wannier center is computed by using a linear combination of the degenerate wavefunctions. The Chern numbers are extracted based on the number of jumps experienced by the Wannier center evolution as a function of θ. By inspecting the two doubly degenerate bands defining the degeneracy at P (**Figure S1b,c**), we note that each pair of degenerate bands exhibit two jumps, which signals that the Chern numbers of the two doubly degenerate bands are -2 and 2, with the sign corresponding to negative or positive jumps. The quadrupole degeneracy at P is therefore a charge-2 Dirac point. In contrast, the Chern numbers of the three bands around the triple degenerate point are 2, 0, and -2, respectively (**Figure S1d-f**), indicating this degenerate point at Γ is a spin-1 Weyl point.

S2. Multifold degenerate points in a gyroid acoustic crystal

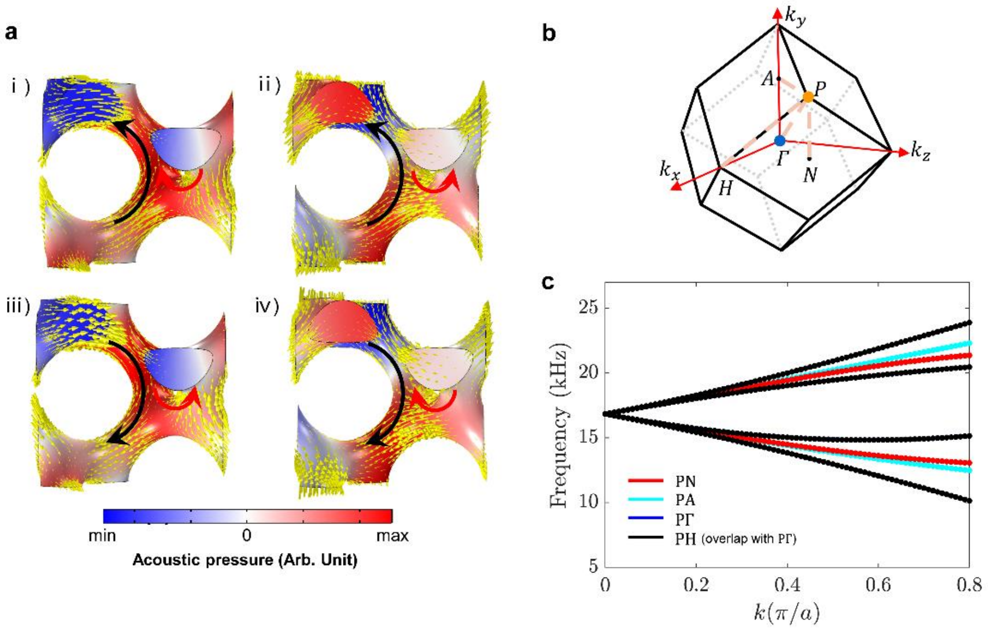

Figure S2. The features of the quadruple degenerate point. a) Four degenerated eigenmodes. The colormap depicts the acoustic pressure and the yellow arrows denote the velocities of the vertexes. The black and red arrows demonstrate the major velocity directions. b) 3D Brillouin zone indicating the symmetry lines crossing at *P*. c) The slope information of the dispersions along the symmetry lines around *P*.

The four eigenmodes of the quadruple degenerate point at *P* are displayed in **Figure S2a**. The colormap depicts the acoustic pressure of the modes and the yellow arrows denote the velocities of the vertexes. The black and red arrows indicate major velocity directions. The degenerate modes i) and iii) exhibit the same acoustic field while the velocity field has opposite chirality (rotation directions). The same features are observed in modes ii) and iv). **Figure S2b** shows the 3D Brillouin zone of the gyroid acoustic crystal and indicates the symmetry lines across at *P*. These bands possess linear dispersion with identical band slopes along with all the directions around the quadruple degenerate point (**Figure S2c**), a feature expected of Dirac points.

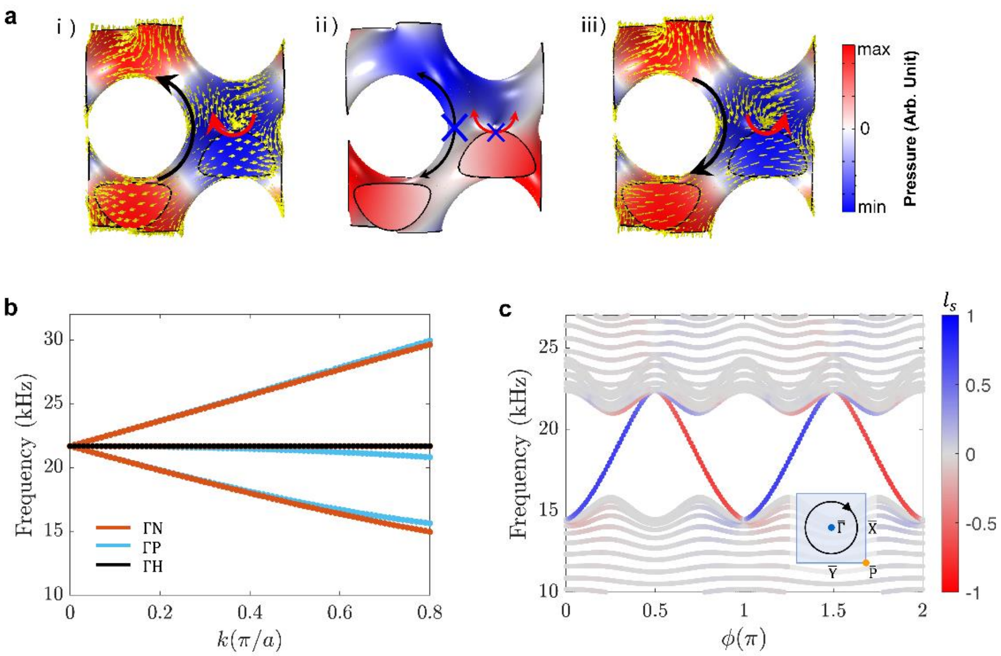

Figure S3. The features of the triple degenerate point. a) Three degenerated eigenmodes. The colormap depicts the acoustic pressure and the yellow arrows denote the velocities of the vertexes. The black and red arrows demonstrate the major velocity directions. b) The slope information of the dispersions along the symmetry lines around *Γ*. c) Surface dispersion along a

circular momentum loop centered at $\bar{\Gamma}$ in surface Brillouin zone. The modes are color-coded by the localization factor l_s.

Figure S3 shows the three eigenmodes of the triple degenerate point. The degenerate modes i) and iii) possess the same acoustic field while the motion velocities have opposite chirality, which is similar to what is described in Figure S2a. Mode ii) has zero velocity field, meaning it is associated with near-zero energy transport due to the flat band. Hence, the three bands passing through the degeneracy at point Γ include two linear dispersion bands and one flat band that has zero or minimal group velocity. The linear dispersion along the symmetry lines around Γ is illustrated in **Figure S3b**. Finally, **Figure S3c** shows the surface dispersion along a circular momentum loop centered at $\bar{\Gamma}$ in surface Brillouin zone, to complement the results shown in the main text corresponding to a loop around $\bar{P}$. The modes are color-coded by the localization factor l_s. The projected dispersion encircling $\bar{\Gamma}$ features the same chiral bands as the dispersion along the loop centered at $\bar{P}$, but with opposite slopes due to the change in reference in momentum space.

S3. Surface dispersions in terms of surface termination

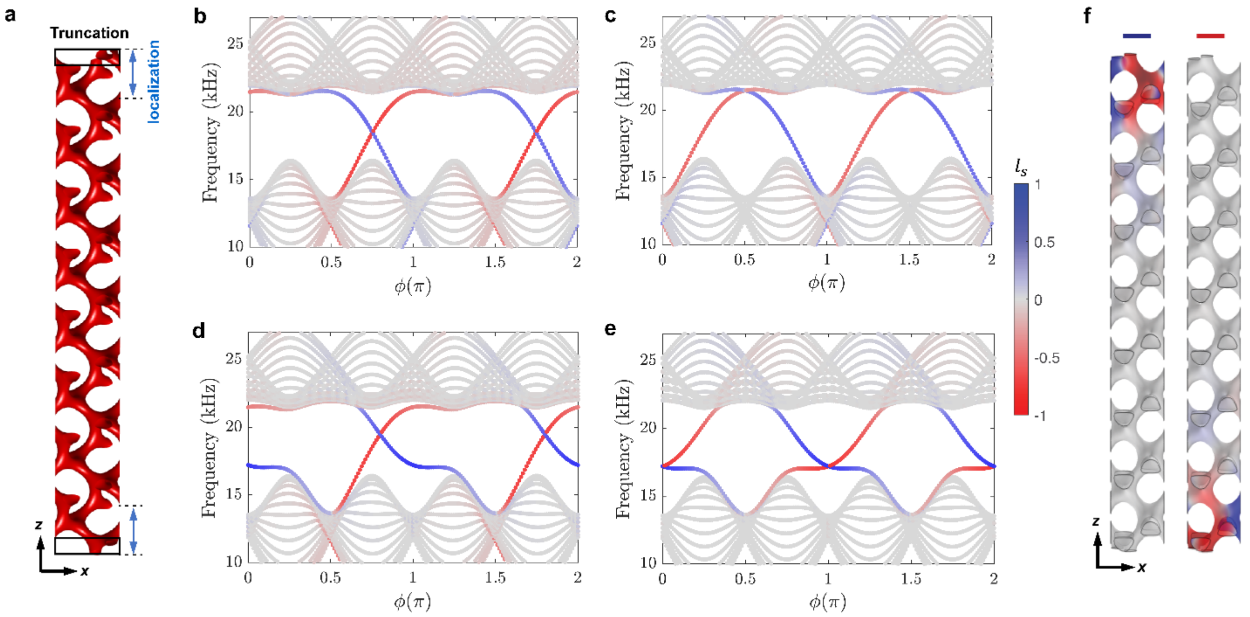

Figure S4. Surface dispersion in terms of surface termination. a) The ribbon structure used in the numerical simulation of surface dispersions. The range marked by the blue arrow indicates the

integration range for surface localization and the range marked by the black box denotes the truncated range of the ribbon structure. b-e) Surface dispersion along the circular loop of radius 0.75π/a centered at $\bar{P}$ with truncation applied at one end, truncation applied at both ends, sound soft boundary applied at one end, and sound soft boundary applied at both ends along the z direction, respectively. The modes are color-coded by the localization factor l_s. f) A demonstration of the surface modes with distinct chirality.

The influence of surface termination on surface dispersions is explored in this session. Here we tune the surface conditions numerically by either truncating or applying soft boundary conditions at the ends of the ribbon structure. **Figure S4a** shows the model of the ribbon structure consisting of 1×1×8 cells with a lattice spacing of 10 mm, where the black boxes denote the range (3 mm) to be removed in the case of a truncated boundary. The surface localization of the modes is identified through a localization factor l_s defined as

$$l_s = \frac{\int_{Top}|p| - \int_{Bottom}|p|}{\int_{Domain}|p|} \qquad \text{(S2)}$$

where the numerator involves the integration of the pressure field at regions around the top and bottom surfaces (see Figure S4a), while the denominator involves the integration along the entire domain of the ribbon. With such a definition, positive and negative l_s values indicate modes localized at the top and bottom surfaces (blue and red colors), while $l_s \approx 0$ indicates a non-localized bulk mode (gray color). The surface dispersions are obtained along the circular loop of radius 0.75π/a centered at $\bar{P}$. **Figure S4b** and **S4c** depict the surface dispersion obtained with the truncation applied at one and both ends, while **Figure S4d** and **S4e** correspond to the cases where sound soft boundary conditions are applied at one and both ends, respectively. Two surface modes at 18 kHz localized at opposite surfaces are displayed in **Figure S4f** as a demonstration. We note that changing the surface termination does not eliminate the existence of surface modes, but their dispersion branches vary. Hence, the symmetry of the gyroid structure results in topological degeneracy which guarantees the existence of chiral surface states for any type of boundary conditions and terminations.

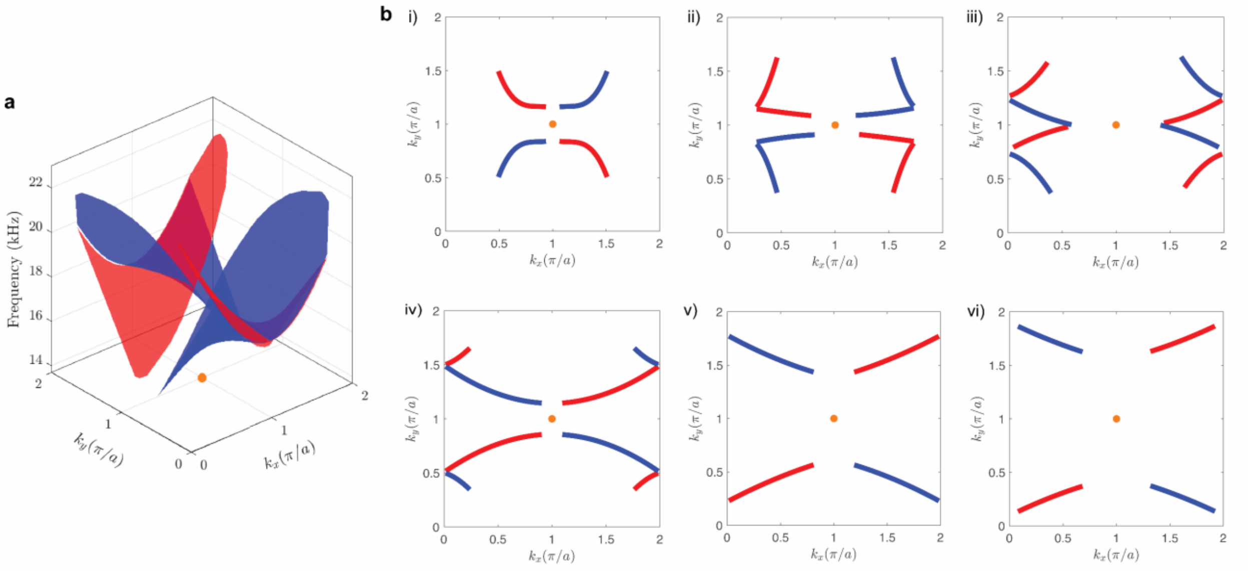

Figure S5. Surface arcs obtained from the gyroid acoustic crystal with soft boundary conditions. a) Dispersion of surface states in the ribbon structure with soft boundary conditions applied on both ends. b) Isofrequency contours of the surface modes selected frequencies. The blue and red modes represent the surface states with positive and negative chirality, respectively.

The surface dispersion of the ribbon structure with soft boundary conditions applied to both surfaces is displayed in **Figure S5** for further visualization of the influence of surface termination on surface modes. The dispersion is mapped on the surface Brillouin zone centered at $\bar{P}$ at the $k_x k_y$ plane. The pattern of the surface sheets here (**Figure S5a**) is different from the case with hard boundary conditions (Figure 2f), and the surface states of opposite chirality intersect along certain momentum lines. The blue and red modes represent the surface states localized at the top and bottom surfaces with positive and negative chirality, respectively. **Figure S5b** shows the isofrequency contours of the surface modes at the frequencies of 16.5 kHz, 17.0 kHz, 17.5 kHz, 18.5 kHz, 20.5 kHz, and 21.5 kHz, respectively. The variation of open arcs in terms of frequency is complicated due to the appearance of twisted surfaces within 17.1~19.8 kHz. These results illustrate how the details of the surface termination can potentially be used as an additional degree of freedom to manipulate the surface states and open arcs.

S4. Numerical observation of acoustic surface modes

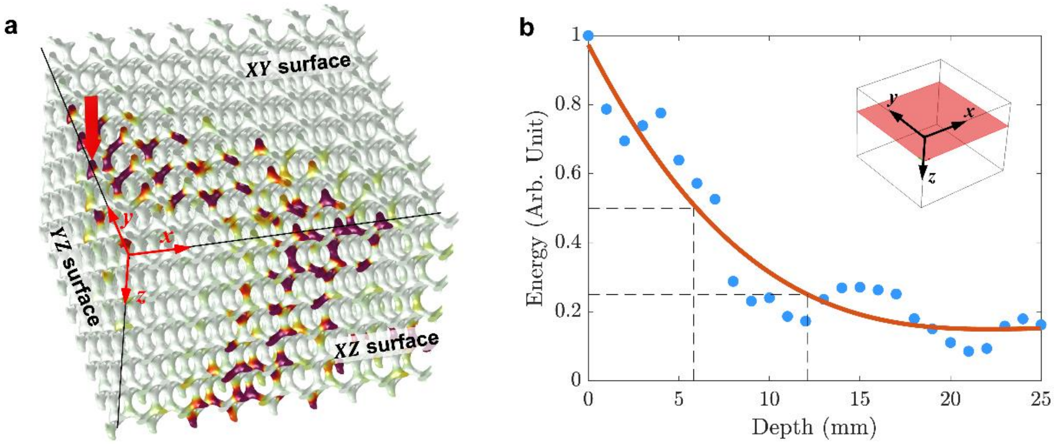

Figure S6. Numerical observation of acoustic surface modes. a) Negative refraction of the acoustic wave with the excitation at the center of the edge separating the XY and YZ surfaces. The red arrow indicates the excitation position. b) The variation of the energy in terms of the depth along the *z* direction. A cross-sectional *xy* plane used for energy integration is marked as a demonstration in the inset.

Figure S6 shows a numerical example of acoustic surface modes with the excitation at the center of the edge separating the XY and the YZ surfaces. Due to limitations in computational power, the model of the acoustic gyroid material consists of $8 \times 8 \times 6$ cells, which is smaller than the experimental sample. The red arrow indicates the excitation position. Similar to the phenomena in Figure 4b, negative refraction of the acoustic wave is observed at 17.2 kHz (**Figure S6a**). The acoustic wave propagates directionally from the XY surface to XZ surface with negative refraction. To further illustrate the features of the excited surface, a sequence of cross-sectional *xy* planes with varying *z* values is selected to explore the change of the energy in terms of the depth along the *z* direction. **Figure S6b** shows the variation of the integrated energy of the cross-sections in terms of depth. The inset shows a cross-section at the *xy* plane as a demonstration. The energy has been normalized based on the maximum energy obtained at the surface. As the dashed lines indicate, in compassion with the energy at the surface (depth is 0 mm), 50% energy is left at the depth ~5.5 mm and 25% energy at the depth ~12 mm, revealing a quick decay of the energy along the depth. Considering the lattice constant is 10 mm, most of the energy is distributed to the surface layer around 1.2 unit cells, signaling the energy is well confined at the surface area of the gyroid acoustic crystal.

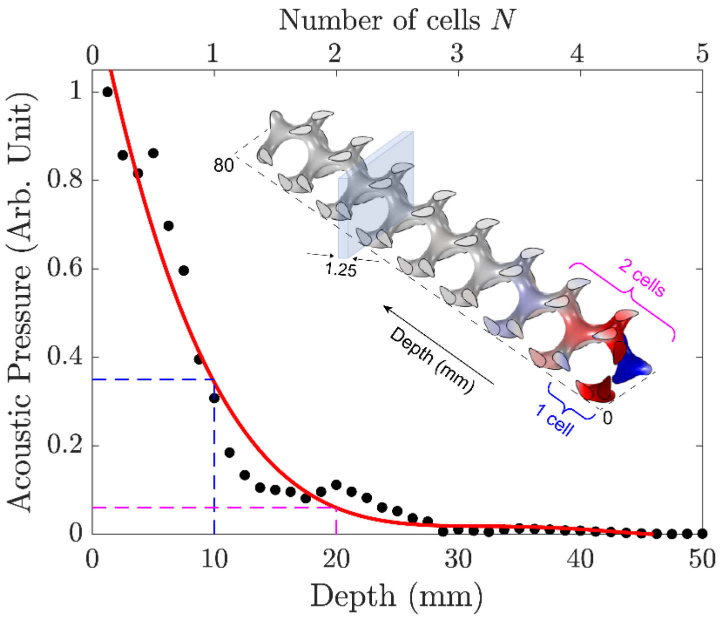

Figure S7. The variation of the acoustic pressure at 18.45 kHz in terms of the depth in a ribbon structure. The inset is the eigenmode with surface localization and the blue box indicates the integration range for the estimation of pressures for a specific depth.

The number of cells in the gyroid acoustic crystal affects two features of the topological states: its degree of localization at the surface, and the ability to observe the propagation of directional waves along the surface. The degree of localization of the surface modes is illustrated in **Figure S7**, which shows the variation of the acoustic pressure in terms of the depth for the ribbon structure described in the main text, and for the eigenmode corresponding to a surface state. The pressure at a specific depth L is approximated by integrating the value of the absolute acoustic pressure within a thin cuboid with a depth within [L-1.25, L] (mm), as the blue box in the inset indicates. As the dashed lines in the figure indicate, ~35% pressure remains at the depth of 10 mm (corresponding to the size of 1 cell), and only 6% pressure remains at the depth of 20 mm (corresponding to the size of 2 cells), signaling the energy is well confined at the first two unit cells of the gyroid acoustic crystal for the surface state. The eigenmode in the inset also evidences this conclusion. Therefore, a gyroid acoustic crystal with $\geq$ 3 cells along each direction is capable to exhibit a localized surface state and we have chosen the value of 6 unit cells along the z direction in our sample (12 $\times$ 12 $\times$ 6), which is sufficient to promote well-localized surface states. However, a larger number of cells (12) is used in the other planes to

properly observe the propagation of waves along the surfaces. We found that this number of cells is sufficient to promote the visualization of the directional waveguiding of the surface state, occurring with minimal wave reflection at the boundaries, and with minimal influence from bulk modes that co-exist in the same frequencies.

References

[1] H. Weng, R. Yu, X. Hu, X. Dai, Z. Fang, *Adv. Phys.* **2015**, *64*, 227;

[2] Y. Yang, H.-x. Sun, J.-p. Xia, H. Xue, Z. Gao, Y. Ge, D. Jia, S.-q. Yuan, Y. Chong, B. Zhang, *Nat. Phys.* **2019**, *15*, 645;

[3] [A. Lau, Symmetry-enriched topological states of matter in insulators and semimetals, Technische Universitaet Dresden, **2017**;

[4] C. Wang, H. Zhang, H. Yuan, J. Zhong, C. Lu, *Front. Optoelectron.* **2020**, *13*, 73.
